# Avian Infective Endocarditis Associated with *Vagococcus fluvialis*: A Case Report and Literature Review

**DOI:** 10.3390/ani16081267

**Published:** 2026-04-21

**Authors:** Ruy D. Chacón, Thamyres Fernandes de Amorim, Tania Cencara Rojas, Karen Tafur-Trujillo, Alexander Ramirez-Montes, Giovanna Sola Castanho, Henrique Lage Hagemann, Julia Ferreira Waldvogel, Claudete S. Astolfi-Ferreira, Andrea Micke Moreno, Antonio J. Piantino Ferreira

**Affiliations:** 1 Department of Pathology, School of Veterinary Medicine and Animal Science, University of São Paulo (USP), São Paulo 05508-270, SP, Brazil; ruychaconv@alumni.usp.br (R.D.C.); gio.sola@yahoo.com.br (G.S.C.); henrique.trick@alumni.usp.br (H.L.H.); julia.waldvogel@usp.br (J.F.W.); csastolfi@gmail.com (C.S.A.-F.); 2 Pathogen Genetics Research Group (PATHO-GEN), OMICS, Lima 15001, Peru; 20191117@lamolina.edu.pe (T.C.R.); tafurtrujillokaren@gmail.com (K.T.-T.); alexanderrozs98@gmail.com (A.R.-M.); 3 Laboratório de Virologia Animal, Departamento de Medicina Veterinária, Universidade Federal Rural de Pernambuco (UFRPE), Recife 52171-900, PE, Brazil; thamyresfamorim@gmail.com; 4 Facultad de Ciencias, Universidad Nacional Agraria La Molina (UNALM), Lima 15024, Peru; 5 Facultad de Ciencias Biológicas, Universidad Nacional de Trujillo (UNT), Trujillo 13011, La Libertad, Peru; 6 Department of Preventive Veterinary Medicine, School of Veterinary Medicine and Animal Science, University of São Paulo (USP), São Paulo 05508-270, SP, Brazil; morenoam@usp.br

**Keywords:** avian infective endocarditis, *Vagococcus fluvialis*, *Enterococcaceae*, multidrug-resistant bacteria, antimicrobial resistance, poultry diseases, MALDI-TOF MS, 16S rRNA

## Abstract

Avian infective endocarditis is an uncommon but severe disease that is typically diagnosed postmortem because of nonspecific clinical signs and rapid progression. In the present study, five broiler chickens (*n* = 5) from a commercial flock were examined with septicemia and lesions suggestive of endocarditis. This study reports the first molecularly confirmed and characterized case of valvular endocarditis associated with multidrug-resistant *Vagococcus fluvialis* in poultry and provides a comprehensive review of bacterial endocarditis in avian species. The case involved a broiler chicken that presented with sudden death and septicemic lesions, including vegetative valvular endocarditis, pericarditis, and multiorgan involvement. Bacterial isolates recovered from cardiac lesions were identified as *V. fluvialis* using MALDI-TOF mass spectrometry and confirmed by 16S rRNA gene sequencing. Antimicrobial susceptibility testing revealed a multidrug resistance profile, with resistance to several antimicrobial classes commonly used in poultry production. The literature review identified published studies describing avian infective endocarditis, which predominantly affects poultry and is caused mainly by Gram-positive bacteria, with clinical signs and necropsy findings largely overlapping across etiologies. These findings highlight the novelty of *V. fluvialis* as a potential etiological agent of avian infective endocarditis and underscores the diagnostic challenges associated with avian endocarditis, particularly when uncommon pathogens are involved, and underscore the importance of advanced identification methods for an accurate etiological determination. Collectively, the results of this study expand the spectrum of bacterial species associated with avian infective endocarditis and emphasize the relevance of antimicrobial resistance and improved diagnostic strategies in poultry health and disease surveillance.

## Simple Summary

Infective endocarditis is a severe and often fatal disease in poultry and wild birds and is usually associated with well-known bacterial pathogens. This study describes a rare case of avian endocarditis caused by a multidrug-resistant strain of *Vagococcus fluvialis*, a bacterium that is seldom reported and frequently overlooked in routine diagnostics. Through detailed pathological, microbiological, and antimicrobial susceptibility analyses, combined with a comprehensive literature review, this work highlights the diagnostic challenges posed by uncommon bacterial agents, the emerging role of antimicrobial resistance, and the potential underestimation of *Vagococcus* species in avian infections. These findings emphasize the importance of accurate identification methods and continuous surveillance to better understand the clinical and epidemiological relevance of rare pathogens in avian diseases.

## 1. Introduction

Infective endocarditis (IE) is a rare but potentially fatal inflammatory disease of the endocardium, the innermost layer of the heart, most often involving one or more cardiac valves [1]. Although the mural endocardium may also be affected in some cases [2], valvular endocarditis remains the predominant presentation in both mammals and birds. In avian species, IE has been documented sporadically, with chickens (*Gallus gallus domesticus*) being the most frequently studied species. Reports in other avian orders, including *Casuariiformes* (*Casuarius casuarius*), *Accipitriformes* (*Aquila chrysaetos*, *Haliaeetus leucocephalus*, and *Buteo jamaicensis*), and *Psittaciformes* (*Anodorhynchus hyacinthinus*, *Ara ararauna*, *Cacatua alba*, *Cacatua galerita*, *Cyanopsitta spixii*, and *Amazona autumnalis salvini*), also exist [3,4,5,6].

In poultry, particularly broiler chickens, IE is still considered uncommon but is of growing concern because of its association with high mortality and economic losses. Diagnosis is typically confirmed *post mortem*, as the disease often lacks specific clinical signs and progresses rapidly [7]. Endocardial lesions are most commonly observed in the left atrioventricular and aortic valves and are typically characterized by vegetative lesions consisting of fibrin, platelets, and bacterial colonies [4]. In birds, septicemia is often the underlying condition that precedes IE, arising from primary infections such as pododermatitis, hepatitis, salpingitis, or even skin wounds [8,9,10]. Disseminated emboli and multiorgan failure are usually the ultimate causes of death rather than valvular dysfunction itself [5].

A wide variety of pathogens, predominantly bacteria, have been implicated in avian IE. Among the Gram-positive organisms, the most frequently isolated include *Streptococcus* spp. (e.g., *S. gallinaceus, S. gallolyticus*), *Enterococcus* spp. (particularly *E. hirae*), *Staphylococcus aureus*, *Erysipelothrix rhusiopathiae*, and *Lactobacillus jensenii* [4,6,7,11]. Gram-negative pathogens include *Pasteurella multocida*, *Avibacterium* spp., *Escherichia coli*, *Pseudomonas aeruginosa*, and *Enterobacter cloacae* [6,12,13]. Additionally, protozoan and viral agents such as *Toxoplasma gondii* and avian reoviruses, respectively, have been associated with cardiac lesions consistent with endocarditis [6].

Among the streptococci, *Streptococcus gallinaceus*, which was first isolated from broiler chickens in the early 2000s, has since been detected in both poultry and swine, suggesting possible cross-species transmission and zoonotic potential [11,14,15]. Similarly, *Enterococcus hirae* has emerged as a major etiological agent in broilers and is responsible for significant mortality during grow-out periods [16]. Interestingly, antimicrobial resistance has also been noted in some of these organisms. For instance, *Streptococcus pluranimalium* strains isolated from pigs in Brazil have been shown to be resistant to several antimicrobial classes, including tetracyclines, fluoroquinolones, macrolides, sulfonamides, clindamycin, and tiamulin, but remain susceptible to β-lactams such as penicillin and ampicillin [17]. This finding highlights the potential impact of selective antimicrobial pressure in livestock systems on the emergence and distribution of IE pathogens.

Despite the growing number of reports describing avian infective endocarditis, the diversity and pathogenic role of uncommon or rarely reported bacterial agents remain poorly understood. The genus *Vagococcus*, which includes Gram-positive cocci that are phylogenetically related to enterococci and lactococci, has rarely been reported in human and animal infections and is almost never associated with endocarditis in birds [18,19,20,21,22]. *Vagococcus fluvialis* (*V. fluvialis*), which was originally isolated from river water, has been recovered from clinical specimens from humans and animals but remains poorly characterized in terms of its pathogenic potential [19,22]. This lack of information highlights an important knowledge gap regarding the potential involvement of the *Vagococcus* genus in avian infective endocarditis. A description of its involvement in IE would represent an unusual and noteworthy clinical event in birds.

Given the broad spectrum of bacterial species implicated in avian infective endocarditis and the increasing importance of early and accurate diagnosis in both clinical and epidemiological settings, the identification of unusual pathogens in cardiac lesions warrants attention. The detection of *V. fluvialis* in a case of valvular endocarditis in a chicken represents a novel finding with potential implications for our understanding of IE pathogenesis, host susceptibility, and environmental reservoirs.

The objective of this study is to present the first molecularly confirmed and characterized case report of valvular endocarditis associated with *V. fluvialis* in poultry, accompanied by a comprehensive review of the literature on bacterial endocarditis in avian species. This report aims to contribute to the veterinary and microbiological knowledge base by expanding the list of potential causative agents, reinforcing the need for improved bacteriological diagnostics, and identifying new targets for surveillance and prevention strategies in poultry health management.

## 2. Case Report

### 2.1. Clinical History and Samples

The present case began in August 2022 on a broiler farm consisting of 30,000 45-day-old birds in the state of Sao Paulo, Brazil. Approximately 1% of the flock exhibited lethargy and depression, ruffled feathers, weakness, and sudden death. According to information provided by the farm, the flock was approaching the scheduled slaughter age and no antimicrobial treatments were administered prior to slaughter. The farm reported routine biosecurity and management practices typical of commercial broiler production systems. Five dead birds were sent to the Laboratory of Avian Diseases, School of Veterinary Medicine, University of São Paulo, for diagnostic investigation. The submission of five birds reflects routine diagnostic sampling practices in Brazilian poultry farms, where a limited number of representative animals are necropsied to investigate the etiology of disease events at the flock level. Because the flock was close to slaughter age, additional flock-level diagnostic procedures or therapeutic interventions were not feasible, and the diagnostic request was primarily intended to identify the etiological agent responsible for the observed lesions and inform corrective measures for subsequent production cycles.

At necropsy, the heart showed marked alterations, with whitish to yellowish vegetative lesions firmly attached to the valves, consistent with valvular endocarditis, and areas of myocardial necrosis. The pericardial sac showed evident signs of pericarditis, with thickening and fibrinous deposits. Splenomegaly and hepatomegaly were noted, and the liver was congested and friable. These cardiac and systemic lesions, together with the generalized congestion of organs, were consistent with septicemia. Additionally, inspectors at the slaughterhouse reported the presence of similar cardiac lesions resulting in condemnation of affected hearts, supporting the occurrence of endocarditis at the flock level.

Representative samples of the valvular lesions were collected aseptically, both indirectly using sterile swabs and directly as tissue fragments during necropsy. These samples were immediately processed for a microbiological analysis by inoculation into Luria–Bertani (LB) broth and incubated at 37 °C under aerobic conditions for 18 h to allow bacterial enrichment prior to isolation. Following enrichment, a loopful of the broth culture was streaked onto Luria–Bertani (LB) agar plates using a sterile inoculating loop and incubated at 37 °C for 18–24 h. After incubation, well-isolated colonies with homogeneous morphology were observed and subsequently used for further identification analyses. Two colonies were obtained after culture on LB agar (Invitrogen, Carlsbad, CA, USA) for 18 h at 37 °C and independently identified (USP-2657/1 and USP 2657/2). This consistent recovery from lesion-derived samples supports a true etiological association rather than environmental or laboratory contamination.

### 2.2. Identification by Mass Spectrometry (MALDI-TOF MS)

Bacterial identification was performed using matrix-assisted laser desorption ionization-time of flight mass spectrometry (MALDI-TOF MS) with a previously established protocol [22]. Briefly, samples were processed according to the reference method and analyzed using a Microflex™ mass spectrometer (Bruker Daltonik GmbH, Bremen, Germany). Spectra were acquired in the mass range of 2–20 kDa with an α-cyano-4-hydroxycinnamic acid (α-cyano) matrix (10 mg/mL in 50% acetonitrile/2.5% trifluoroacetic acid; Bruker Daltonik GmbH). For identification, MALDI BioTyper™ software version 3.0 was employed. Two replicates of each sample were spotted into separate wells of the plate, and each replicate was analyzed twice. The resulting spectra were compared against the reference library from the manufacturer, and standard Bruker interpretative criteria were applied. A score ≥ 2.0 was considered indicative of species-level identification, whereas scores between 1.7 and 2.0 were considered suggestive of genus-level identification. The results revealed that both original colonies were identified as *V. fluvialis*, with score values of 2.33 (USP-2657/1) and 2.39 (USP-2657/2), respectively.

### 2.3. Identification by 16S rRNA Sequencing and Phylogenetic Analysis

The same pure colonies processed for MALDI-TOF MS analysis (USP-2657/1 and USP 2657/2) were subcultured in LB broth (Invitrogen, Carlsbad, CA, USA) for 18 h at 37 °C for DNA extraction using a DNeasy^®^ Blood & Tissue Kit (Qiagen, Hilden, Germany) according to the manufacturer’s recommendations. The DNA suspension was quantified with a NanoDrop One (Thermo Scientific^TM^, Wilmington, DE, USA) and stored at −80 °C until use in subsequent procedures.

The 16S rRNA gene was amplified with the universal primers 27F (5′-AGAGTTTGATCCTGGCTCAG-3′) and 1492R (5′-GGTTACCTTGTTACGACTT-3′), and the PCR conditions were consistent with those reported by Xenoulis et al. [23]. PCR products were purified with a QIAquick^®^ Gel Extraction Kit (Qiagen, Hilden, Germany) and sequenced using the Sanger method on a 3500xL Genetic Analyzer with the BigDye^TM^ Terminator v3.1 Cycle Sequencing Kit (Applied Biosystems, Carlsbad, CA, USA).

Sequencing products were assembled with Geneious Prime^®^ 2020.2.4 (www.geneious.com). Following an initial BLAST (https://blast.ncbi.nlm.nih.gov/Blast.cgi, accessed on 16 April 2026) search to confirm the genus and potential species, which showed complete sequence identity with *V. fluvialis* reference sequences (100%), a phylogenetic analysis was conducted by comparing the partial sequence against reference sequences of the *Vagococcus* genus and other genera from the *Enterococcaceae* family obtained from the GenBank database. To root the phylogenetic tree, sequences from *Tetragenococcus muriaticus* and *Bavariicoccus seileri* were included as outgroups in the analysis. Multiple sequence alignment was performed with an iterative refinement method (FFT-NS-i) on MAFFT v7.48 [24]. The best-fit substitution model for the phylogenetic analysis was estimated using the Bayesian information criterion (BIC), which selected the K80 + R model. The construction of phylogenetic trees was performed with PhyML v3.0 [25] with the maximum likelihood method, and nodes were assessed using Shimodaira–Hasegawa (SH)-like branch support and edited with iTOL v6.4 [26]. Pairwise sequence identity scores and matrices for nucleotides were estimated with the SDT v1.2 tool [27].

**Figure 1:**
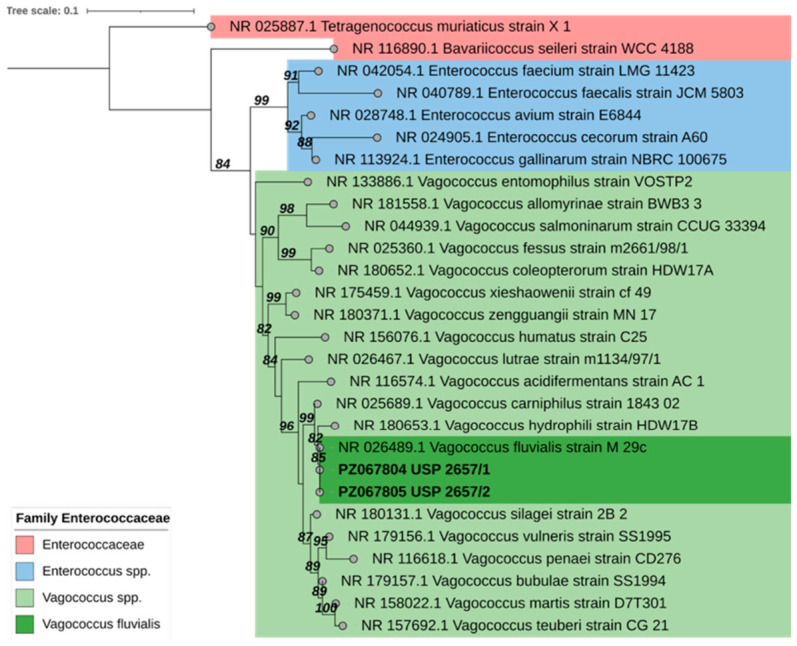
Phylogenetic tree of partial 16S rRNA sequences (1433 bp) for the *Enterococcaceae* family, highlighting the *Vagococcus* genus. The scale bar represents the number of base substitutions per site. The sequences obtained in this study are highlighted in bold.

The results indicated that the USP-2657/1 and USP 2657/2 isolates clustered together with *V. fluvialis* strain M 29c and were closer to the other *Vagococcus* sp. reference strains (Figure 1). The assembled sequences were deposited in GenBank under accession numbers PZ067804 (USP 2657/1) and PZ067805 (USP 2657/2).

**Figure 2:**
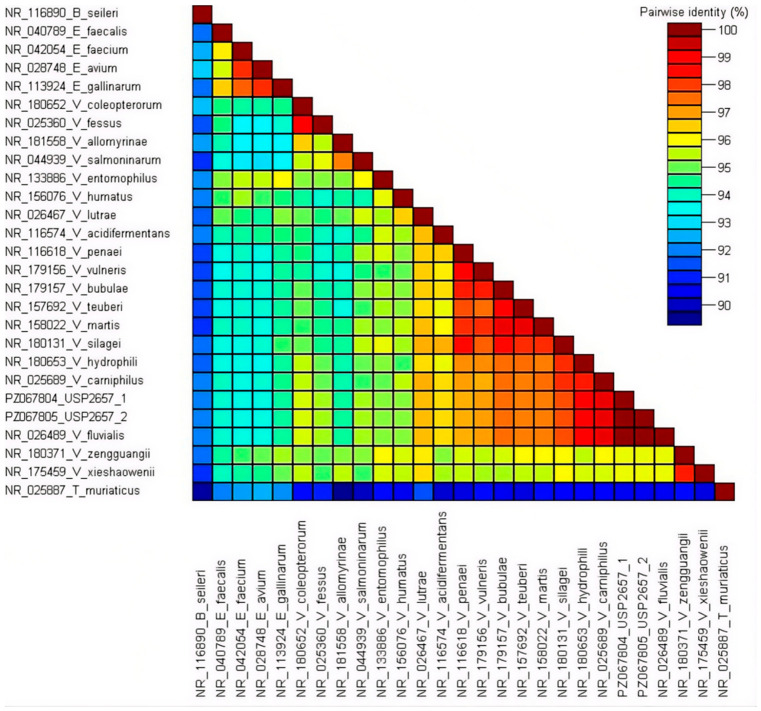
Pairwise nucleotide identity of partial 16S rRNA sequences (1433 bp) for the *Enterococcaceae* family. The values are visualized as color ranges.

The pairwise identity analysis of partial 16S rRNA sequences for the *Enterococcaceae* family revealed the total identity (100%) of the USP-2657/1 and USP-2657/2 isolates with respect to the *V. fluvialis* reference strain M 29c and among themselves (Figure 2). Nucleotide identity with other members of the *Vagococcus* genus ranged from 93.6% to 99.3%. Nucleotide identity with other members of the *Enterococcaceae* family ranged from 88.9% to 93.4%. Although 16S rRNA gene sequencing may have limited resolution for closely related taxa within the *Enterococcaceae* family, species identification in this study was robustly supported by concordant phylogenetic clustering and high nucleotide identity with reference *V. fluvialis* sequences.

### 2.4. Antimicrobial Resistance Profile Obtained by Determining the Minimal Inhibitory Concentrations (MICs)

The minimal inhibitory concentrations (MICs) of antibiotics against the USP-2657/1 and USP-2657/2 isolates were determined using the broth microdilution technique. This method employed commercially prepared Sensititre™ Standard Susceptibility MIC Plates (TREK Diagnostic Systems/Thermo Fisher Scientific, Waltham, MA, USA) and adhered to the guidelines outlined in the fifth edition of the CLSI document VET01 [28].

Brain heart infusion (BHI) broth supplemented with 5% fetal bovine serum (FBS) served as the culture medium. After an incubation at 37 °C for 24 h, the culture was adjusted to a turbidity equivalent to the 0.5 McFarland standard and verified using a spectrophotometer. This suspension was then diluted 1:1000 in Mueller–Hinton II broth supplemented with 5% FBS to achieve the final inoculum for the MIC assay.

Fifty microliters (µL) of the final inoculum was dispensed into each well of the microplate. The plate was incubated at 37 °C for 24 h. The MIC was visually determined as the lowest concentration of antibiotic in the well that exhibited no visible growth (absence of button formation). The *Streptococcus pneumoniae* ATCC 49619 strain was included as a quality control measure to ensure the accuracy of the MIC determination. Due to the lack of established breakpoints for *Vagococcus* sp. in CLSI documents [28], antimicrobial susceptibility is presented as MIC profiles, highlighting the distribution of MIC values for each tested antibiotic.

Multidrug resistance (MDR) is defined as resistance to three or more antimicrobial classes. Antimicrobial susceptibility testing of USP-2657/1 and USP-2657/2 isolates revealed significant multidrug resistance (MDR) profiles for both isolates (Table 1). Both isolates were consistently resistant to marbofloxacin, oxytetracycline, spectinomycin, streptomycin, sulfamethoxazole, tiamulin, and tildipirosin. In addition, USP-2657/1 also presented resistance to clindamycin, enrofloxacin, and trimethoprim/sulfamethoxazole. On the other hand, USP-2657/2 also presented resistance to cephalexin and gentamicin. These results indicate common resistance to at least seven different antimicrobial classes, such as fluoroquinolones, tetracyclines, aminocyclitols, aminoglycosides, sulfonamides, pleuromutilins, and macrolides.

### 2.5. Case Summary

The findings of this case confirm the occurrence of valvular endocarditis associated with multidrug-resistant *V. fluvialis* in broiler chickens approaching slaughter age. The diagnosis was supported by characteristic gross lesions and microbiological identification using MALDI-TOF MS and 16S rRNA sequencing. No antimicrobial treatment was applied prior to slaughter, and additional flock-level diagnostics were not feasible due to the production stage. The presence of similar cardiac lesions at the slaughterhouse, leading to condemnation of affected hearts, supports the occurrence of the condition at the flock level. This case highlights the involvement of an uncommon bacterial agent in avian infective endocarditis and underscores the importance of comprehensive etiological investigation to inform control strategies in poultry production systems.

## 3. Literature Review

For this review, we searched the PubMed database using the combined terms “endocarditis”, “broiler”, “layer”, “breeder”, “chicken”, “poultry”, “bird”, and “avian”. The search strategy was based on the following Boolean expression: ((endocarditis[Title/Abstract]) AND (broiler[Title/Abstract])) OR ((endocarditis[Title/Abstract]) AND (layer[Title/Abstract])) OR ((endocarditis[Title/Abstract]) AND (breeder[Title/Abstract])) OR ((endocarditis[Title/Abstract]) AND (chicken[Title/Abstract])) OR ((endocarditis[Title/Abstract]) AND (poultry[Title/Abstract])) OR ((endocarditis[Title/Abstract]) AND (bird[Title/Abstract])) OR ((endocarditis[Title/Abstract]) AND (avian[Title/Abstract])). The search covered the period from 1 January 1950 to 30 June 2025 and retrieved a total of 158 records. After identifying relevant and available manuscripts, we examined the references cited within each selected article and included those meeting our inclusion criteria, specifically those that described clinical information and associated bacteria in the study. Studies were included if they reported cases of infective endocarditis in avian species, provided accessible full-text information, and described bacterial identification associated with the lesions. Studies not meeting these criteria were excluded. The filtered studies were categorized into two groups based on the avian populations that were analyzed: domestic birds and wild birds. From each study, clinical signs and necropsy findings were extracted and standardized to ensure consistent terminology and avoid semantic redundancy. These results were then grouped into categories based on the affected system, including nonspecific signs. The frequency of each sign and necropsy finding, as well as each category, was calculated separately for poultry and wild birds. The number of studies retained after each selection step is detailed in the following sections. The findings for each group are presented below.

### 3.1. Endocarditis in Poultry

In the case of poultry, the literature review and filtering process identified 21 studies related to endocarditis, and the data obtained from the case report in the present study were added, resulting in a total of 22 studies. The information gathered is presented in Table 2.

The distribution of infective endocarditis cases across poultry types was broad and was detected almost exclusively in chickens, with only a single case reported in meat-type ducks aged 1–2 weeks. Eight cases of IE were identified in broilers ranging from 6 to 58 days of age. In layers, only three reports were found, with ages within standard production ranges (20–61 weeks). Among the breeder broilers, nine cases were recorded within typical breeding ages (20–61 weeks), along with two exceptional cases: one involving breeders between 2–6 weeks of age and another involving a 7-year-old breeder bird.

The included studies identified a total of 14 different bacterial species, with a high frequency of the family *Enterococcaceae* and particularly the species *Enterococcus hirae* (6/14, 42.86%). These bacteria were distributed across five main families: *Enterococcaceae* (*E. hirae*, *E. faecalis*, *E. faecium*, and *V. fluvialis*), *Streptococcaceae* (*S. equi*, *S. gallinaceus*, *S. gallolyticus*, and *S. pluranimalium*), *Staphylococcaceae* (*S. aureus*, *S. agnetis*, and *S. simulans*), *Pasteurellaceae* (*Avibacterium gallinarum* and *A. endocarditidis*), and *Helcococcaceae* (*Helcococcus ovis*, one case). Moreover, certain pathogens showed associations with specific production categories: *Enterococcus hirae* was detected in broilers and young birds; *Streptococcus equi* subsp. *zooepidemicus* was detected in layers; and *Avibacterium endocarditidis* and *Staphylococcus agnetis* were detected in breeders. These observations suggest that the type of poultry production may influence the epidemiology of IE, as different bacterial species appear to be associated with particular production systems or bird categories.

**Figure 3:**
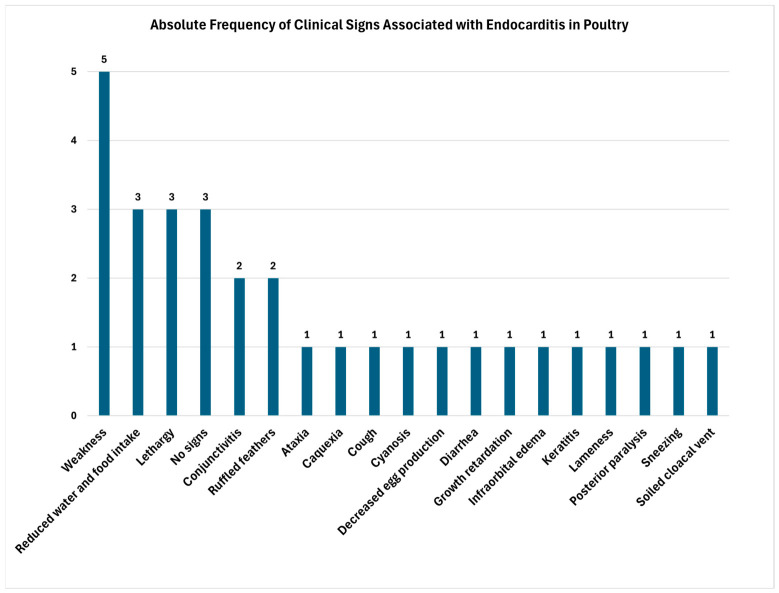
Absolute frequencies of clinical signs associated with endocarditis in poultry.

The clinical signs of endocarditis reported in the literature are highly variable and frequently nonspecific, reflecting both cardiac and systemic involvement caused by the etiologic agent [11]. As shown in Figure 3, clinical presentation in poultry is dominated by nonspecific and systemic signs, rather than organ-specific indicators. Although weakness, reduced water and feed intake, and lethargy were commonly reported [13,32,40], these findings are not distinctive and overlap with multiple infectious conditions. Notably, some studies reported the absence of clinical signs, indicating that subclinical endocarditis was diagnosed only through necropsy and a histopathological examination [35,36]. Additionally, a consistently high frequency of mortality was observed across studies (Table 2), underscoring the clinical and economic impact of the condition. Overall, the predominance of nonspecific clinical manifestations is evident [11,30,31,32,33,34,35,36,37,38,39,40,41,42,43,44,45].

**Figure 4:**
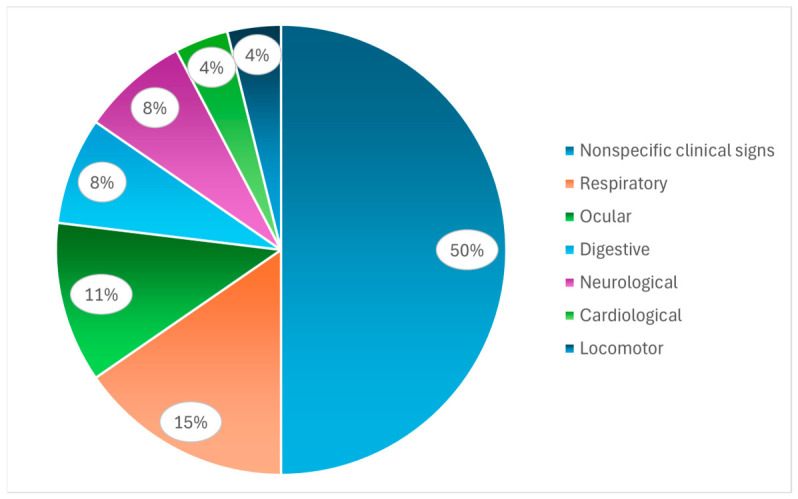
Relative frequencies of the categories of clinical signs associated with endocarditis in poultry.

When categorized by system, the predominance of nonspecific signs becomes more evident (Figure 4). Although respiratory, ocular, digestive, and neurological manifestations were reported [7,13,16,29,41], none of these categories predominated strongly, reinforcing the absence of pathognomonic clinical patterns. These findings indicate that clinical presentation alone is insufficient for a definitive diagnosis, as similar signs are shared across a wide range of infectious diseases caused by infectious bronchitis virus, avian influenza virus, Newcastle disease virus, infectious laryngotracheitis virus, avian metapneumovirus, avian pathogenic *Escherichia coli*, *Avibacterium paragallinarum*, *Salmonella* spp., *Clostridium* spp., *Campylobacter* spp., *Mycoplasma* spp., *Chlamydia* spp., *Aspergillus* spp., coccidia, as well as enteric viruses such as astrovirus, rotavirus, reovirus, parvovirus, avian nephritis virus, and adenovirus, as well as noninfectious conditions such as nutritional disorders and intoxications [46,47,48,49,50,51]. Thus, the overlap of clinical signs across systems highlights the need for complementary diagnostic approaches. The low frequency of reported cardiac signs in poultry does not indicate their actual absence; rather it reflects the challenges and limitations associated with handling and individual examination in flock management under field conditions [52].

**Figure 5:**
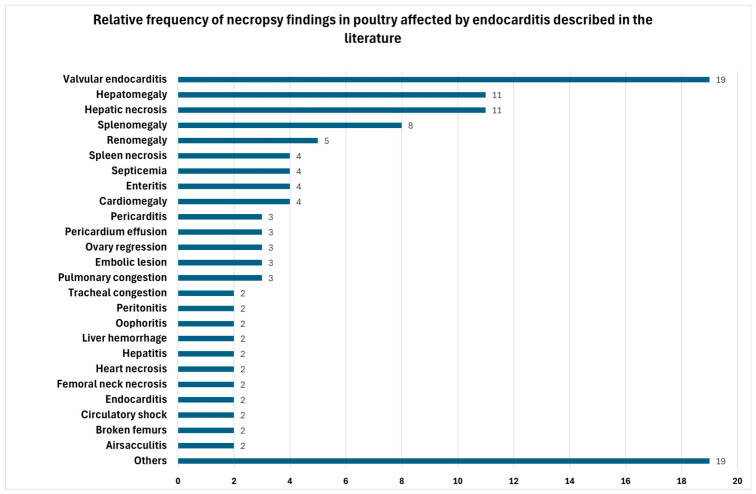
Absolute frequencies of necropsy findings associated with endocarditis in poultry.

Among the necropsy findings, the cardiovascular system was the most frequently affected, with valvular endocarditis being the most common lesion (Figure 5). This consistent distribution of lesions confirms the central role of cardiac involvement in avian IE, even when clinical signs are absent or nonspecific. These findings reinforce the importance of performing a necropsy associated with a histopathological examination, since in most birds, no clinical signs suggestive of cardiac involvement were observed, despite the high frequency of cardiac lesions identified postmortem. In chickens, endocarditis is most commonly observed in the atrioventricular valves [29,30,35,41]. In addition to endocarditis itself, the most frequent cardiac lesions include cardiomegaly, pericarditis, pericardial effusion, and myocardial necrosis [7,13,16,29,31,33,34,45].

**Figure 6:**
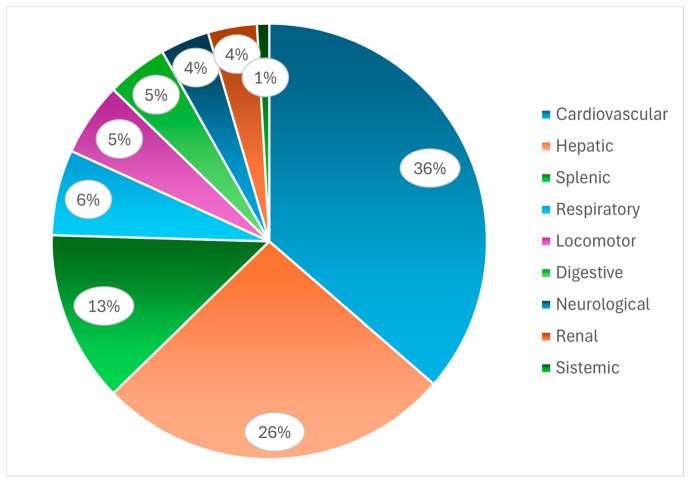
Relative frequency of different categories of necropsy findings associated with endocarditis in poultry.

In addition to the cardiovascular system, the tissues that showed the highest frequency of lesions were the liver, spleen, and respiratory system (Figure 6). The main hepatic and splenic lesions were necrosis and enlargement of these organs (hepatomegaly and splenomegaly) [11,31,32,33,34,40,42,44,45]. In the respiratory system, the predominant lesions observed were pneumonia and pulmonary congestion [13,45].

Lesions affecting the nervous system were among the least frequently reported, whereas renal involvement was less common but clinically relevant. Renal findings were consistent with previously described clinical manifestations and included mainly nephromegaly, renal degeneration, and necrosis [11,16,32,33,34]. In contrast, clinical manifestations involving the nervous system were rare, with reported lesions primarily consisting of inflammatory and hemorrhagic changes [30].

### 3.2. Endocarditis in Wild Birds

The literature review and filtering process identified 8 studies related to endocarditis in wild birds. The information gathered from these studies is presented in Table 3.

The distribution of reported IE cases in wild birds was dispersed across several taxonomic groups, with a predominance of psittacines (Order *Psittaciformes*; Families *Psittacidae* and *Cacatuidae*), followed by raptors (Order *Accipitriformes*; Family *Accipitridae*) and a single case in a ratite (Order *Casuariiformes*; Family *Casuariidae*). Reported cases included macaws (*Ara ararauna*, *Anodorhynchus hyacinthinus*), parrots (*Amazona autumnalis salvini*, *Cyanopsitta spixii*), and cockatoos (*Cacatua alba*) ranging from 6 to 40 years of age, as well as raptors such as *Buteo jamaicensis*, *Aquila chrysaetos*, and *Haliaeetus leucocephalus*, including adults and individuals up to 27 years old. The studies identified a limited number of bacterial species, with *Staphylococcus aureus* being the most frequently isolated pathogen, and additional cases involving *Enterobacter cloacae*, *Lactobacillus jensenii*, and *Enterococcus casseliflavus*. These pathogens belong to four bacterial families: *Enterobacteriaceae*, *Staphylococcaceae*, *Enterococcaceae*, and *Lactobacillaceae*. Notably, the recurrent isolation of *Staphylococcus aureus* occurred in older psittacines and raptors, whereas the case of *Casuarius casuarius* highlights the susceptibility of large terrestrial ratites.

**Figure 7:**
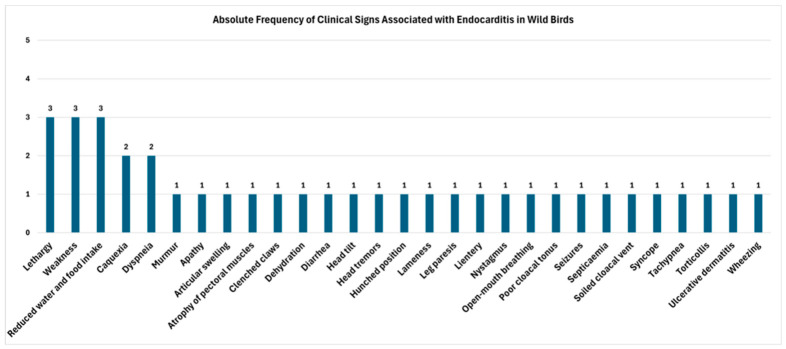
Absolute frequencies of clinical signs associated with endocarditis in wild birds.

The clinical manifestations of IE in wild birds were more diverse but less frequently reported than in poultry. This greater diversity likely reflects both species variability and the more individualized clinical observation of wild birds compared to flock-based production systems. Nevertheless, several similarities were identified, particularly regarding the most frequent clinical signs, such as increased mortality, followed by lethargy and weakness (Figure 7) [5,53,54,55,56,57,58,59]. Other reported findings included cachexia, decreased water and food consumption, dyspnea, and cardiac murmurs [53,56,57,59].

**Figure 8:**
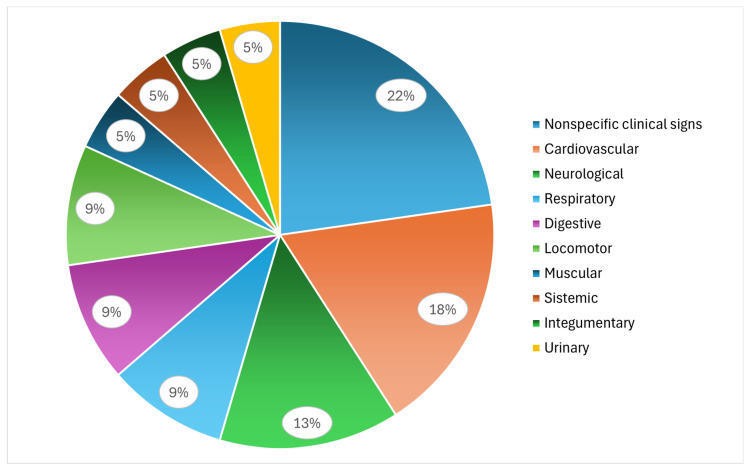
Relative frequencies of the categories of clinical signs associated with endocarditis in wild birds.

In wild birds, nonspecific signs also predominated (Figure 8). However, clinical signs related to specific systems were more frequently represented, particularly those related to the neurological, respiratory, locomotor, and digestive systems, as well as a higher proportion of cardiac signs than in poultry. As similarly observed in poultry, a high frequency of mortality was also reported in wild birds. Overall, wild birds exhibited a greater diversity of clinical manifestations.

Regarding respiratory signs, dyspnea was the most frequently reported sign in wild birds, consistent with the progression of cardiac diseases that may evolve into secondary respiratory compromise [53,59].

Neurological signs were also more frequent in wild birds, with reported manifestations including ataxia, tremors, seizures, and nystagmus [59]. Like in poultry, locomotor signs included lameness, paralysis, and traumatic limb injury, whereas among the digestive signs, diarrhea was most commonly observed [58].

Among the less frequent findings in wild birds were signs related to the muscular, urinary, and integumentary systems, including pectoral muscle atrophy, urate staining of feathers, ulcerative dermatitis, and septicemia [53,55]. Although less common, these manifestations should also be considered in the differential diagnosis of suspected endocarditis.

Cardiac signs were more frequently reported in wild birds than in poultry, likely reflecting more individualized clinical observations. The most commonly described findings were arrhythmias and cardiac murmurs [53,59].

**Figure 9:**
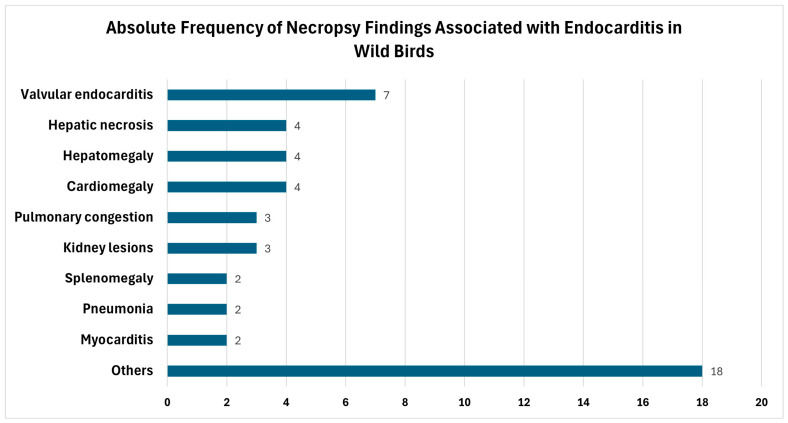
Absolute frequencies of necropsy findings associated with endocarditis in wild birds.

Among the necropsy findings, the cardiovascular system was also the most frequently affected system in wild birds (Figure 9). Valvular endocarditis was the most common lesion and was most frequently observed in the aortic valves [5,56,58,59]. Other detectable cardiovascular pathologies included cardiomegaly and myocarditis [53,54,56,57,58,59].

**Figure 10:**
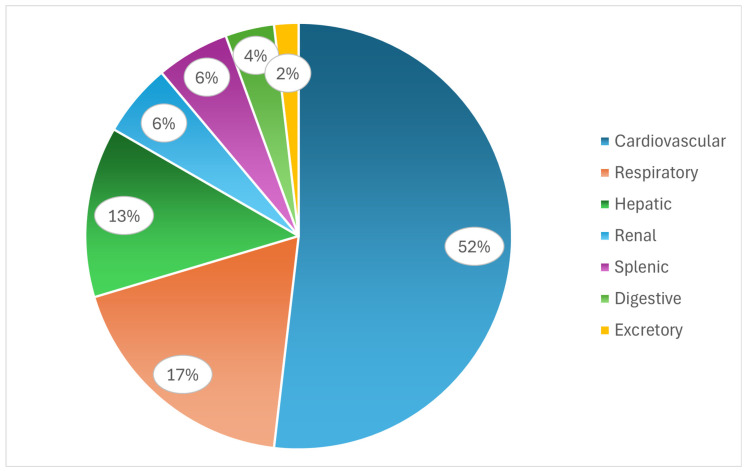
Relative frequencies of different categories of necropsy findings associated with endocarditis in wild birds.

In addition to the cardiovascular system, when categorized, the respiratory system (mainly pulmonary congestion and pneumonia) and the hepatic system (primarily hepatomegaly and hepatic necrosis) were the next most frequently affected at necropsy (Figure 10) [53,54,55,56,57,58,59]. Kidney lesions and splenomegaly were also reported in more than one study [53,54,55,56].

### 3.3. Bacterial Species Associated with Avian Infective Endocarditis and Their Occurrence in Humans

We evaluated the potential implications of bacteria associated with infective endocarditis (IE) in birds for human health by conducting a targeted search of reported cases and the available literature. As a result, the majority of bacterial species identified in avian IE have also been reported as causative agents of IE in humans. Table 4 summarizes the bacterial families and species associated with avian endocarditis that have also been documented in human infective endocarditis to contextualize these findings within a broader One Health framework.

This comparative analysis showed that almost all bacterial families associated with avian IE have also been reported in human cases. Notably, the families *Streptococcaceae* and *Enterococcaceae*, which also include the greatest species diversity, have documented reports of causing IE in humans for all listed species.

In contrast, three avian IE-associated species have not been reported as causes of infective endocarditis in humans. *Avibacterium endocarditidis* is a previously described species linked to valvular endocarditis in chickens, with no evidence of causing human infection to date [12,13,37]. Similarly, *Helcococcus ovis* has been recognized in humans as an opportunistic pathogen, with reports of ocular infections and detection at various sites in the body, particularly in the context of dysbiosis or polymicrobial infections, suggesting a potential zoonotic relevance rather than a proven role in endocarditis [83]. In addition, *Staphylococcus agnetis* has not been implicated in human infections nor is it considered part of the human microbiota; however, it has been reported to produce bacteriocins with activity against *Staphylococcus aureus*, the leading cause of infective endocarditis in humans [44,84].

Specifically, this study reports the first molecularly confirmed and characterized case of infective endocarditis (IE) in birds associated with *V. fluvialis*. Although apparently less frequent than other bacterial agents, *V. fluvialis* has been identified in a wide range of animal hosts [17,18]. In humans, reports of IE caused by *V. fluvialis* are limited to only two published studies [20,21]. However, several additional studies have documented its presence in the bloodstream or its isolation from blood cultures [19,85]. Furthermore, *V. fluvialis* has been detected in a variety of other human infectious clinical contexts [86,87,88].

The pathogenic bacteria included in this review are known to harbor a diverse arsenal of virulence genes that contribute to their infectious potential, several of which are particularly relevant in the context of infective endocarditis (IE). Among the major categories of virulence factors are those that increase adhesion to host cells, promote biofilm formation, and interfere with host coagulation mechanisms [86,89,90]. Adhesion to extracellular matrix proteins such as fibronectin, collagen, and laminin represents a critical step in bacterial colonization and the development of endocarditis. Bacteria express specific adhesins that mediate these interactions, facilitating biofilm formation and immune evasion [91,92]. Representative examples include clumping factor A (ClfA), fibronectin-binding protein A (FnBPA), extracellular adhesion protein (Eap), and the coagulases von Willebrand factor-binding protein (vWbp) and coagulase (Coa) in *S. aureus* [84]. In *Enterococcaceae*, particularly *E. faecalis* (which is taxonomically closer to *V. fluvialis*), a specific set of virulence factors with a role in IE has been highlighted [89]. These virulence factors include proteins involved in bacterial aggregation (aggregation substances Asa1, Asc10, and Asp1), a hemolysin, cell wall glycolipids, general stress protein 24 (Gls24), endocarditis- and biofilm-associated pili (Ebp), gelatinase (GelE), adhesin to collagen (Ace), enterococcal fibronectin-binding protein A (EfbA), and the membrane metalloprotease Eep [89].

For *V. fluvialis*, no studies have directly linked the expression of specific genes to its ability to cause IE. Nevertheless, genomic analyses have revealed the presence of numerous homologous genes annotated with virulence-associated functions, including adhesion factors, secretion systems, and iron acquisition mechanisms [93,94]. Although many of these genes are associated with relatively low virulence, their presence suggests a relevant pathogenic potential, particularly in hospital environments or in immunocompromised hosts [92]. One such factor shared with *V. fluvialis* homologs is EfbA, which plays a key role in bacterial adhesion to the extracellular matrix and in biofilm development. In *E. faecalis*, EfbA mediates binding to fibronectin and collagen, and its deletion significantly reduces virulence and biofilm formation in endocarditis models. Moreover, immunization with EfbA confers protection against infection in both avian and human hosts, underscoring its central role in pathogenesis [95]. Additional genes, such as *atlA* (autolysin) and *groEL* (chaperonin), have also been associated with adhesion and biofilm formation in Gram-positive bacteria [94].

Overall, these findings support the potential zoonotic relevance of avian IE-associated pathogens and reinforce the importance of integrating veterinary and human health perspectives when evaluating emerging or uncommon etiological agents.

## 4. Discussion

Infective endocarditis in birds remains an underrecognized but clinically significant condition, particularly within intensive poultry production systems. Although traditionally regarded as rare, accumulating evidence from case reports and outbreak descriptions indicates that avian IE may be more prevalent than is currently appreciated, largely because of its nonspecific clinical presentation and frequent postmortem diagnosis. The present case of valvular endocarditis caused by *V. fluvialis*, combined with a comprehensive review of previously reported avian cases, reinforces the relevance of this disease as an indicator of systemic bacterial infection, management deficiencies, and emerging pathogenic threats in poultry populations. Importantly, the identification of an unusual and multidrug-resistant bacterial species highlights the need to expand the spectrum of microorganisms considered in avian endocarditis and move beyond classical etiological paradigms.

Across the reviewed literature, avian infective endocarditis consistently emerges as a disease characterized by generalized and nonspecific clinical signs, which substantially hinder early recognition at the flock level. Rather than specific diagnostic indicators, the clinical presentation reflects a systemic septicemic process, in which cardiovascular involvement becomes evident only at advanced stages. This lack of pathognomonic signs explains why a clinical suspicion of endocarditis is rarely raised *ante mortem*. Similarly, necropsy findings, although more informative, tend to reflect an advanced systemic disease rather than isolated cardiac dysfunction. Taken together, these observations support the interpretation of avian endocarditis as the consequence of sustained bacteremia, leading to the colonization of cardiac valves under conditions of endothelial damage and altered hemodynamics [10].

The diagnostic challenges associated with avian infective endocarditis are further compounded by the limitations of conventional bacteriological identification methods, particularly when uncommon or fastidious organisms are involved. As illustrated by the present case, *V. fluvialis* may be easily misidentified or overlooked when the analysis relies solely on phenotypic characteristics or routine biochemical assays, given its phylogenetic proximity to enterococci and lactococci [19,96]. The incorporation of advanced diagnostic tools such as MALDI-TOF MS and 16S rRNA gene sequencing has proven essential for accurate species-level identification, enabling the detection of rare pathogens that would otherwise remain unrecognized [13,41,44,57]. Beyond taxonomic resolution, these approaches are critical for uncovering hidden diversity among opportunistic pathogens and for refining our understanding of host–pathogen interactions in avian systems. The improved diagnostic resolution not only enhances case reporting accuracy but also contributes to a more realistic understanding of pathogen diversity in avian endocarditis, with direct implications for epidemiological surveillance and comparative pathology.

Antimicrobial resistance represents an additional and increasingly concerning dimension of avian infective endocarditis. The multidrug resistance profile observed in the *V. fluvialis* isolates described here is consistent with other studies of IE reported for several bacterial species implicated in avian septicemia and endocarditis. This pattern suggests that antimicrobial resistance in avian IE is not restricted to well-recognized pathogens, but may also be present in underreported or emerging bacterial species. Resistance to multiple antimicrobial classes, including tetracyclines, macrolides, aminoglycosides, and fluoroquinolones, reflects the selective pressure exerted by antimicrobial use in animal production systems [7,13,31,53,54,58]. Notably, the emergence of resistance in rarely reported bacteria challenges the assumption that antimicrobial resistance is confined to well-known pathogens and highlights the role of lesser-known species as potential reservoirs of resistance determinants. From a clinical perspective, multidrug resistance further limits therapeutic options and may contribute to treatment failure, delayed intervention, and increased mortality in affected flocks [96,97,98].

From a pathogenesis perspective, the involvement of *V. fluvialis* in valvular endocarditis may be explained by mechanisms analogous to those described for other Gram-positive cocci, including adhesion to damaged endothelium, biofilm formation, and persistence within fibrin–platelet aggregates [99]. Although specific virulence factors in *Vagococcus* spp. remain poorly characterized, its phylogenetic proximity to enterococci suggests the potential presence of adhesins, extracellular matrix-binding proteins, and immune evasion strategies that facilitate the colonization of cardiac tissues [100,101]. In addition, the observed multidrug resistance phenotype may contribute to prolonged bacteremia by reducing bacterial clearance, thereby increasing the likelihood of cardiac valve colonization. These hypotheses warrant further validation through genomic and functional studies.

In addition to its veterinary relevance, avian IE caused by uncommon bacteria raises important questions regarding the zoonotic risk and One Health implications. Several bacterial species traditionally associated with avian endocarditis, including streptococci and enterococci, are well-established human pathogens, and sporadic reports of genetically related strains in animals and humans suggest possible cross-species transmission or shared environmental reservoirs. Although *V. fluvialis* has rarely been implicated in human infections, its documented presence in clinical samples, combined with its demonstrated virulence traits and antimicrobial resistance, supports cautious consideration of its zoonotic potential. The convergence of antimicrobial resistance, environmental persistence, and potential host adaptability reinforces the importance of a One Health approach, integrating animal, human, and environmental surveillance systems to monitor emerging pathogens. Poultry workers, veterinarians, and slaughterhouse personnel may be exposed to such organisms through direct contact or environmental contamination, emphasizing the importance of integrated surveillance approaches that bridge the animal and human health sectors.

Finally, the findings presented in this work point toward several future research directions that are essential for advancing knowledge on avian IE. Further studies are needed to elucidate the virulence mechanisms underlying cardiac colonization and systemic dissemination by uncommon bacterial species, including the roles of adhesins, biofilm formation, and host–pathogen interactions. Expanded genomic analyses may provide insights into antimicrobial resistance determinants, mobile genetic elements, and evolutionary relationships among isolates from different hosts and environments. In parallel, large-scale epidemiological investigations are needed to determine the true prevalence of avian endocarditis and to assess its effects on animal welfare and production efficiency. Collectively, these efforts will contribute to improved diagnostic accuracy, more effective prevention strategies, and a deeper understanding of the complex interactions between pathogens, hosts, and antimicrobial use in avian systems.

## 5. Conclusions

This study documents the first molecularly confirmed and characterized case of avian infective endocarditis caused by multidrug-resistant *V. fluvialis* and integrates this finding within a comprehensive review of the literature on bacterial endocarditis in birds. The results demonstrate that avian infective endocarditis is a multifactorial and frequently underdiagnosed condition that is often detected only at advanced stages because of nonspecific clinical signs and generalized pathological findings. These findings support the importance of performing routine necropsies and the inclusion of cardiac evaluation during necropsy routines, even in the absence of specific clinical signs. The identification of an uncommon bacterial species reinforces the need to broaden the etiological framework traditionally associated with this disease and highlights the importance of the implementation of laboratory diagnosis whenever possible, considering rare or overlooked pathogens in cases of septicemia and cardiac lesions in poultry.

Furthermore, the multidrug resistance profile observed in the isolates underscores the growing relevance of antimicrobial resistance in avian pathology, even among infrequently recognized bacteria. Beyond the implementation of laboratory diagnosis, antimicrobial susceptibility testing should be incorporated into routine diagnostics to guide therapeutic decisions and monitor emerging resistance patterns. These approaches should be integrated into structured surveillance programs at the farm and processing levels to enable the early detection and continuous monitoring of antimicrobial resistance trends. The use of molecular biology (e.g., PCR or MALDI-TOF) is also recommended, particularly in atypical or recurrent cases. Accurate species-level identification using advanced diagnostic tools is essential to avoid misclassification and support effective epidemiological surveillance. In practical terms, the adoption of combined diagnostic workflows, including necropsy, microbiological culture, MALDI-TOF MS, and molecular confirmation, should be encouraged in veterinary diagnostic laboratories and poultry production systems. Collectively, these findings emphasize the need for integrated diagnostic, pathological, and microbiological approaches to improve the detection, management, and prevention of avian infective endocarditis while strengthening antimicrobial stewardship and surveillance strategies in poultry production systems.

## Figures and Tables

**Table 1:** MIC ranges and breakpoints applied to *Vagococcus fluvialis* isolates *.

Antimicrobial	MIC Range (µg/mL)	S	I	R	USP-2657/1 MIC		USP-2657/2 MIC	
Amoxicillin/Clavulanate	1/0.5–32/64	≤8/4	16/8	≥32/16	≤1/0.5	S	≤1/0.5	S
Ampicillin	1–64	≤8	16	≥32	≤1	S	≤1	S
Ceftiofur	0.25–8	≤2	4	≥8	≤0.25	S	≤0.25	S
Cephalexin	0.5–32	≤2	4	≥8	4	I	32	R
Clindamycin	0.25–4	≤0.5	1–2	≥4	>4	R	0.5	S
Enrofloxacin	0.25–4	≤0.5	1–2	≥4	>4	R	2	I
Florfenicol	0.5–8	≤4	8	≥16	4	S	4	S
Gentamicin	0.5–16	≤4	8	≥16	8	I	16	R
Marbofloxacin	0.06–4	≤1	2	≥4	4	R	4	R
Oxytetracycline	2–32	≤4	8	≥16	>32	R	>32	R
Spectinomycin	8–128	≤32	64	≥128	>128	R	>128	R
Streptomycin	1–16	-	-	≥8	>16	R	>16	R
Sulfamethoxazole	128–512	≤256	-	≥512	512	R	512	R
Tiamulin	2–32	≤16	-	≥32	32	R	>32	R
Tildipirosin	1–64	≤4	8	≥16	>16	R	>16	R
Trimethoprim/Sulfamethoxazole	1/9–4/76	≤2/38	-	≥4/76	4/76	R	≤1/9	S
Tulathromycin	1–64	≤16	32	≥64	4	S	16	S

* Isolates were classified as susceptible (S), intermediate (I), or resistant (R).

**Table 2:** Reported cases of infective endocarditis in poultry: pathogens, clinical signs, and necropsy findings.

Year/Ref.	Bird ^A^	Age	Associated Bacteria	Clinical Signs/Mortality	Necropsy Findings
1966 [29]	Layer	30 weeks	*Streptococcus equi* subsp. *zooepidemicus*	Cyanosis; diarrhea; decreased egg production; increased mortality	Catarrhal enteritis; congested lung; enteritis; friable liver; friable spleen; hemoperitoneum; hemorrhagic spleen; hemothorax; hepatitis; hepatomegaly; liver necrosis; pericardial effusion; septicemia; valvular endocarditis
1971 [30]	n.s.	adults	*Enterococcus faecalis*	Sudden death	Bacterial embolic lesions; CNS hemorrhage; CNS infarcts; intracerebral inflammatory foci; leptomeningitis; valvular endocarditis
1988 [31]	Meat-type *	1–2 weeks	*Streptococcus faecium*	Increased mortality	Airsacculitis; endocarditis; hepatomegaly; pericarditis; septicemia; splenomegaly
1995 [32]	Layer	52 weeks	*Avibacterium gallinarum*	Reduced water and feed intake; increased mortality; weakness	Hepatomegaly; renomegaly; splenomegaly; valvular endocarditis
2004 [11]	Breeder	26 weeks	*Streptococcus gallinaceus*	Increased mortality	General congestion; liver hemorrhage; liver necrosis; myocardial necrosis; ovarian regression; renomegaly; spleen hemorrhage; spleen necrosis; valvular endocarditis
2005 [33]	Broiler	6–33 days	*Enterococcus hirae*	Increased mortality	Hepatomegaly; pericarditis; renomegaly; splenomegaly
2005 [34]	Broiler	4 weeks	*Enterococcus hirae*, *Streptococcus gallinaceus*	Increased mortality	Hepatomegaly; liver necrosis; myocardium necrosis; necrotic enteritis; pericarditis; renomegaly; spleen necrosis; splenomegaly; valvular and mural endocarditis
2008 [35]	Broiler	53–58 days	*Streptococcus gallolyticus subsp. gallolyticus*	No signs	Spleen necrosis; valvular endocarditis
2010 [36]	Breeder	n.s.	*Enterococcus faecalis*	no signs	Septicemia; valvular endocarditis
2010 [37]	Breeder	44–61 weeks	*Avibacterium endocarditidis*, *Enterococcus faecalis*, *Staphylococcus aureus*, *Streptococcus pluranimalium*	Increased mortality	Hepatomegaly; regressive ovary; valvular endocarditis
2011 [16]	Broiler	n.s.	*Enterococcus hirae*	Increased mortality; posterior paralysis	Cardiomegaly; cartilage damage at the femoral head; femoral neck necrosis; liver hemorrhage; liver necrosis; renomegaly; thrombotic lesions; valvular endocarditis
2011 [38]	Broiler	2 weeks	*Enterococcus hirae*	Increased mortality	Congestion and edema of the lungs; valvular endocarditis
2013 [39]	Layer	45 weeks	*Streptococcus equi* subsp. *zooepidemicus*	Cachexia; lethargy; soiled cloacal vent	Airsacculitis; bronchopneumonia; liver necrosis; oophoritis/peritonitis; spleen necrosis; valvular endocarditis
2014 [40]	Breeder	58 weeks	*Avibacterium endocarditidis*	Reduced water and feed intake; ruffled feathers; weakness	Hepatomegaly; liver necrosis; regression of the ovary; splenomegaly; valvular endocarditis
2017 [41]	Breeder	7 years	*Helcococcus ovis*	Ataxia; lethargy	Distended intestine; hepatic necrosis; hepatomegaly; cardiac necrosis; spherical mass on the testicle; valvular endocarditis
2017 [42]	Breeder	20 weeks	*Staphylococcus agnetis*	Increased mortality	Circulatory shock; hepatomegaly; liver necrosis; septicemia; splenomegaly
2017 [7]	Broiler	30 days	*Staphylococcus simulans*	Increased mortality; lameness	Cardiomegaly; cartilage damage at the femoral head; femoral neck necrosis; liver necrosis; valvular endocarditis
2019 [43]	Broiler	n.s.	*Enterococcus hirae*	Increased mortality	n.s.
2020 [44]	Breeder	40 weeks	*Staphylococcus agnetis*	Increased mortality	Circulatory shock; hepatomegaly; liver necrosis; splenomegaly
2024 [13]	Breeder	30–42 weeks	*Avibacterium endocarditidis*	Conjunctivitis; cough; reduced water and feed intake; increased mortality; keratitis; sneezing; infraorbital edema	Catarrhal enteritis; congestion and edema of the liver; epicardial/cardiac hemorrhage; mural endocarditis; oophoritis; pericardium effusion; pericardial effusion; peritonitis; pulmonary congestion; tracheal congestion; valvular endocarditis
2025 [45]	Breeder	2–6 weeks	*Enterococcus hirae*	Growth retardation; increased mortality	Ascites; broken femurs; cardiomegaly; embolic lesions (arteria ischiadica); embolic lesions (arteria pulmonalis); hepatomegaly; hepatitis; splenomegaly; valvular endocarditis
2025 [this study]	Broiler	45 days	*Vagococcus fluvialis*	Lethargy; ruffled feathers; sudden death; weakness	Friable liver; cardiac necrosis; hepatomegaly; pericarditis; septicemia; splenomegaly; valvular endocarditis

^A^ All birds correspond to *Gallus gallus domesticus*, while * indicates *Anas platyrhynchos domesticus*. n.s. = not specified. CNS = central nervous system.

**Table 3:** Reported cases of infective endocarditis in wild birds: pathogens, clinical signs, and necropsy findings.

Year/Ref.	Bird	Age	Pathogen	Clinical Signs/Mortality	Necropsy Findings
1992 [53]	*Ara ararauna*	6 years	*Enterobacter cloacae*	Reduced water and feed intake; atrophy of the pectoral muscles; cachexia; dehydration; dyspnea; lethargy; murmur; tachypnea; weakness	Cardiomegaly; hepatomegaly; kidney lesions; splenomegaly
2009 [54]	*Amazona autumnalis salvini*	30 years	*Lactobacillus jensenii*	Apathy; clenched claws; head tilt; weakness	Endocarditis; presence of a friable grey-brown mass in the right ventricle; interstitial pneumonia; kidney lesions; liver necrosis; myocarditis; pneumonia; thickened endocardium; ventriculitis; proventriculitis
2012 [5]	*Buteo jamaicensis*	n.s.	*Staphylococcus* sp.	Traumatic lesion to the cubital joint; mortality	Valvular endocarditis
2014 [55]	*Anodorhynchus hyacinthinus*	18 years	*Staphylococcus aureus*	Mortality; ulcerative dermatitis	Distension of the duodenum; hepatomegaly; increased pericardial fluid volume; kidney lesions; myofiber necrosis; pericardial thickening; pleural thickening; pulmonary congestion
2019 [56]	*Cacatua alba*	15 years	*Staphylococcus aureus*	Reduced water and feed intake; lethargy; leg paresis; poor cloacal tone; soiled cloacal vent; weakness	Dilatation of the urodeum and proctodeum; epicarditis; hepatomegaly; liver necrosis; myocarditis; splenomegaly; valvular endocarditis
2021 [57]	*Cyanopsitta spixii*	40 years	n.s.	Cachexia; mortality; septicemia	Air sac hemorrhage; arterial lesions; cardiomegaly; chronic hepatopathy; hepatic necrosis; transmural hemorrhage in the heart; pulmonary congestion; pulmonary emphysema
2023 [58]	*Casuarius casuarius*	22 years	*Enterococcus casseliflavus*	Diarrhea; dysorexia; hunched position; lameness; lethargy; lientery	Cardiomegaly; gall bladder distension; hepatomegaly; liver necrosis; pulmonary congestion; spleen necrosis; valvular endocarditis
2023 [59]	*Aquila chrysaetos*,	adult	*Staphylococcus aureus*	Dyspnea; head tremors; nystagmus; syncope; torticollis	Endocardial hemorrhage; valvular endocarditis; pulmonary adenocarcinoma with metastasis
2023 [59]	*Haliaeetus leucocephalus*	27 years	n.s.	Mortality	Valvular endocarditis

n.s. = not specified.

**Table 4:** Bacterial species associated with infective endocarditis in humans reported in the literature.

Family	Species	Reports on Human IE ^1^
*Streptococcaceae*	*Streptococcus equi* subsp. *zooepidemicus*	[60,61]
	*Streptococcus gallinaceus*	[62,63]
	*Streptococcus gallolyticus* subsp. *gallolyticus*	[64,65]
	*Streptococcus pluranimalium*	[66,67]
*Enterococcaceae*	*Enterococcus casseliflavus*	[68,69]
	*Enterococcus faecalis*	[70,71]
	*Enterococcus hirae*	[72,73]
	*Vagococcus fluvialis*	[20,21]
*Staphylococcaceae*	*Staphylococcus agnetis*	n.f.
	*Staphylococcus aureus*	[74,75]
	*Staphylococcus simulans*	[76,77]
*Pasteurellaceae*	*Avibacterium endocarditidis*	n.f.
	*Avibacterium gallinarum*	[78]
*Enterobacteriaceae*	*Enterobacter cloacae*	[79,80]
*Peptoniphilaceae*	*Helcococcus ovis*	n.f.
*Lactobacillaceae*	*Lactobacillus jensenii*	[81,82]

^1^ I.E.: infective endocarditis. n.f.: not found.

## Data Availability

The assembled sequences were deposited in GenBank under accession numbers PZ067804 (USP 2657/1) to PZ067805 (USP 2657/2).
